# GFPuv-Expressing Recombinant Rickettsia typhi: a Useful Tool for the Study of Pathogenesis and CD8^+^ T Cell Immunology in R. typhi Infection

**DOI:** 10.1128/IAI.00156-17

**Published:** 2017-05-23

**Authors:** Matthias Hauptmann, Nicole Burkhardt, Ulrike Munderloh, Svenja Kuehl, Ulricke Richardt, Susanne Krasemann, Kristin Hartmann, Till Krech, Bernhard Fleischer, Christian Keller, Anke Osterloh

**Affiliations:** a Department of Immunology, Bernhard Nocht Institute for Tropical Medicine, Hamburg, Germany; b Department of Entomology, University of Minnesota, St. Paul, Minnesota, USA; c Institute for Neuropathology, University Medical Center Eppendorf, Hamburg, Germany; d Institute for Pathology, University Medical Center Eppendorf, Hamburg, Germany; e Institute of Virology, University Hospital Giessen and Marburg, Marburg, Germany; Yale University School of Medicine

**Keywords:** *Rickettsia typhi*, transformation, GFPuv, CD8^+^ T cell response

## Abstract

Rickettsia typhi is the causative agent of endemic typhus, a disease with increasing incidence worldwide that can be fatal. Because of its obligate intracellular life style, genetic manipulation of the pathogen is difficult. Nonetheless, in recent years, genetic manipulation tools have been successfully applied to rickettsiae. We describe here for the first time the transformation of R. typhi with the pRAM18dRGA plasmid that originally derives from Rickettsia amblyommatis and encodes the expression of GFPuv (green fluorescent protein with maximal fluorescence when excited by UV light). Transformed R. typhi (R. typhi^GFPuv^) bacteria are viable, replicate with kinetics similar to those of wild-type R. typhi in cell culture, and stably maintain the plasmid and GFPuv expression under antibiotic treatment *in vitro* and *in vivo* during infection of mice. CB17 SCID mice infected with R. typhi^GFPuv^ succumb to the infection with kinetics similar to those for animals infected with wild-type R. typhi and develop comparable pathology and bacterial loads in the organs, demonstrating that the plasmid does not influence pathogenicity. In the spleen and liver of infected CB17 SCID mice, the bacteria are detectable by immunofluorescence microscopy in neutrophils and macrophages by histological staining. Finally, we show for the first time that transformed rickettsiae can be used for the detection of CD8^+^ T cell responses. GFP-specific restimulation of spleen cells from R. typhi^GFPuv^-infected BALB/c mice elicits gamma interferon (IFN-γ), tumor necrosis factor alpha (TNF-α), and interleukin 2 (IL-2) secretion by CD8^+^ T cells. Thus, R. typhi^GFPuv^ bacteria are a novel, potent tool to study infection with the pathogen *in vitro* and *in vivo* and the immune response to these bacteria.

## INTRODUCTION

Rickettsia typhi is an obligate intracellular bacterium and the causative agent of endemic typhus, an emerging disease that occurs worldwide. The genus Rickettsiae belongs to the family Rickettsiaceae and is divided into four major groups: the spotted fever group (SFG), which contains the vast majority of known rickettsiae (e.g., Rickettsia rickettsii and Rickettsia conorii), the typhus group (TG) (R. typhi and Rickettsia prowazekii), the transitional group (Rickettsia felis, Rickettsia akari, and Rickettsia australis), and the ancestral group (Rickettsia bellii and Rickettsia canadensis) (1–3).

Although genetic manipulation of rickettsiae has been reported, it remains difficult due to their intracellular life cycle and is far from routine. In addition, selection of transformed TG rickettsiae is problematic because R. typhi and R. prowazekii do not form plaques in standard *in vitro* cell cultures employing L929 fibroblasts. Transposon systems have been used for random knockout of chromosomal genes in R. prowazekii (4–6) facilitated by use of a rifampin selection marker. Transposon mutagenesis has also been successfully applied to R. rickettsii (6, 7) and Rickettsia monacensis transformed to express GFPuv (green fluorescent protein with maximal fluorescence when excited by UV light) and a chloramphenicol resistance marker (8). Furthermore, targeted gene knockout by homologous recombination has been achieved in R. prowazekii (9) and using the targetron system in R. rickettsii (10).

For a long time, plasmids have not been detected in rickettsiae, but it has now become clear that many rickettsial species contain extrachromosomal DNA. Plasmids have been identified in members of the transitional group (R. felis and R. akari), the ancestral group (R. bellii), SFG rickettsiae (Rickettsia amblyommatis, Rickettsia rhipicephali, Rickettsia monacensis, Rickettsia helvetica, Rickettsia massiliae, and Rickettsia hoogstraalii), and Rickettsia buchneri, the rickettsial endosymbiont of Ixodes scapularis (11–14), but seem to be absent in Rickettsia parkeri, Rickettsia montanensis, R. rickettsii, R. conorii, R. typhi, and R. prowazekii (14). Successful transformation and maintenance of plasmids in ancestral (R. bellii), TG, and SFG rickettsiae, including those that do not naturally harbor plasmids (R. prowazekii, R. parkeri, R. montanensis, and R. conorii), has been reported (15–17). In TG rickettsiae, plasmids have not yet been identified, and it is still unclear whether these bacteria carry naturally occurring extrachromosomal DNA or might at all support its presence after experimental introduction. However, plasmid transformation of R. prowazekii was successful (17). The plasmid used in these studies (pRAM18dRGA) originally derives from R. amblyommatis, replicates extrachromosomally, and carries the gene encoding the expression of GFPuv and a rifampin selection marker under the control of the R. rickettsii ompA promoter (15).

In the current study, we successfully used this plasmid for the transformation of R. typhi and generated GFPuv-expressing bacteria (R. typhi^GFPuv^). The bacteria were viable *in vitro* and maintained high plasmid copy numbers (18.5 ± 2.9 copies per bacterium) under rifampin selection *in vitro*. Moreover, the plasmid did not alter pathogenicity. Transformed R. typhi bacteria caused a comparable course of disease with pathology similar to that of their wild-type counterparts in susceptible CB17 SCID mice and reached bacterial loads comparable to those of wild-type R. typhi in the organs. R. typhi^GFPuv^ bacteria were detectable in macrophages and neutrophils in the spleens of infected mice by immunofluorescence microscopy of histological sections. Moreover, we show for the first time that GFP-expressing transformed rickettsiae can be used for the analysis of rickettsia-specific CD8^+^ T cell responses. Spleen cells from R. typhi^GFPuv^-infected BALB/c mice reacted to major histocompatibility complex class II (MHC-II)-deficient antigen-presenting cells that either were infected with R. typhi^GFPuv^, expressed cytosolic GFP, or were pulsed with a CD8^+^ GFP epitope peptide with the production of gamma interferon (IFN-γ), interleukin 2 (IL-2), and tumor necrosis factor alpha (TNF-α). Thus, R. typhi^GFPuv^ bacteria represent a useful tool for the study of R. typhi infection *in vitro* and *in vivo*, as well as of the immune response to these bacteria.

## RESULTS

### Generation and detection of GFPuv-expressing recombinant R. typhi by immunofluorescence microscopy and flow cytometry.

**FIG 1:**
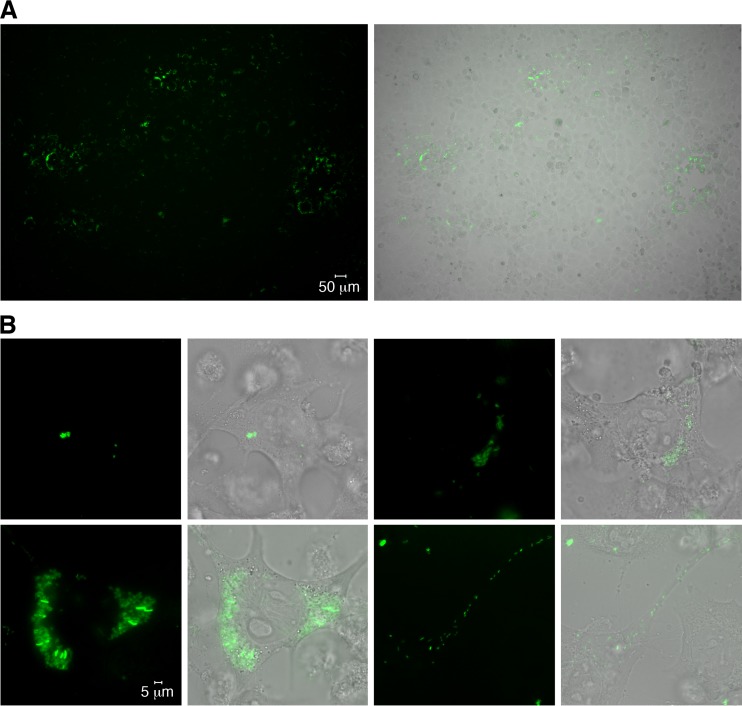
Detection of R. typhi^GFPuv^ in infected L929 cell cultures by fluorescence microscopy. (A) Cell culture flasks (25 cm^2^) with irradiated L929 cells were inoculated with R. typhi^GFPuv^ and analyzed by fluorescence microscopy 5 days after infection. Foci of infected cells are visible. (B) For higher-resolution images, L929 cells were infected with R. typhi^GFPuv^ in Ibidi slides and analyzed 48 h after infection. Images of four cells at different stages of infection in the same cell culture are shown at the same magnification. Invading R. typhi bacteria moved to the nucleus (top left) and replicated in close proximity to the nucleus (top right and bottom left). In some cells, R. typhi bacteria appeared to move into cell protuberances, probably to leave the cell (bottom right).

Successful transformation of purified R. typhi with the GFPuv-encoding plasmid pRAM18dRGA was achieved using 2.4 μg plasmid DNA, 18-kV/cm field strength, and the instrument-inherent capacity and resistance values (10 μF and 600 Ω), which resulted in a pulse duration of 5.7 ms. Nonirradiated L929 cells were infected with electroporated R. typhi, and transformed bacteria were selected by the addition of 10 ng/ml rifampin to the culture medium 24 h after inoculation. With this protocol, recombinant R. typhi^GFPuv^ bacteria were detectable within 9 days postinfection by fluorescence microscopy. Figure 1A shows a representative image of an L929 cell culture infected with R. typhi^GFPuv^. Foci of infected cells in cell culture flasks are visible (Fig. 1A). Higher-resolution live images were taken from R. typhi^GFPuv^-infected L929 cells plated on Ibidi slides. Figure 1B shows single cells at different stages of infection in a single cell culture. R. typhi particles that had entered the cell moved to the nucleus (Fig. 1B, top left), and clusters of replicating bacteria were consistently found in close proximity to the nucleus (Fig. 1B, top right and bottom left). In a few cells, bacteria moved through cell protuberances and seemed to leave the cell (Fig. 1B, bottom right). These data show that R. typhi^GFPuv^ can be easily detected by immunofluorescence microscopy and can be used for *in vivo* imaging studies.

**FIG 2:**
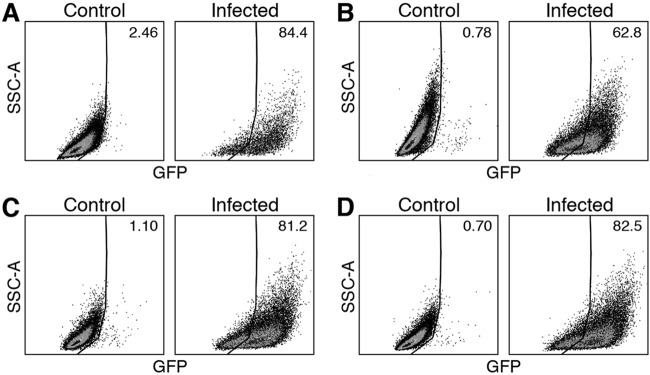
Detection of R. typhi in L929 cells by flow cytometry employing different methods of fixation. R. typhi^GFPuv^-infected L929 cells were fixed in 2% PFA (A), FoxP3 staining buffer (B), Cytofix/Cytoperm solution (C), or formaldehyde-methanol (D) after 6 days of culture and analyzed by flow cytometry (GFP, *x* axis; side scattered light area [SSC-A], *y* axis). Uninfected L929 cells were used as a control. The numbers indicate the percentages of R. typhi^GFPuv^-positive cells.

We further analyzed R. typhi^GFPuv^-infected cells by flow cytometry. Various methods of fixation that are commonly used for intracellular staining of immune cells and that are known to abolish infectivity of the bacteria, which is necessary for removal of specimens from biosafety level 3 (BSL3) containment, were studied. L929 cells were infected with R. typhi^GFPuv^ and analyzed after 6 days of culture. At that time, approximately 80% of the cells were positive for R. typhi^GFPuv^ with 2% paraformaldehyde (PFA) fixation (Fig. 2A), fixation in Cytofix/Cytoperm solutions (Fig. 2C), or FoxP3 staining buffer (Fig. 2D). The lowest percentage of R. typhi^GFPuv^-positive cells was measured after formaldehyde-methanol fixation (62.8%) (Fig. 2B). These data show that R. typhi^GFPuv^-infected cells are detectable by flow cytometry when treated with appropriate fixatives.

### Recombinant R. typhi^GFPuv^ bacteria are viable and stably express the transgene in the presence of rifampin.

**FIG 3:**
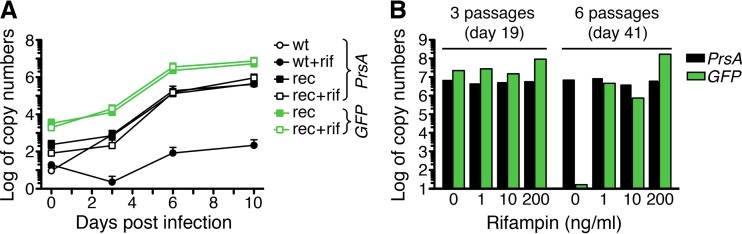
R. typhi^GFPuv^ bacteria are viable and stably express the *gfp* transgene in the presence of rifampin. (A) Tissue culture flasks (25 cm^2^) with irradiated L929 cells were inoculated with comparable numbers of R. typhi^GFPuv^ (rec) (*n* = 2) and wild-type R. typhi (wt) (*n* = 2) bacteria (0.5 copies per cell). The cells were cultured in the presence or absence of 10 ng/ml rifampin (rif). R. typhi prsA-specific (*PrsA*) and *gfp*-specific (*GFP*) qPCRs were performed at the indicated time points (*x* axis) with 10 ng of cell culture DNA. The *y* axis shows the log_10_ copy numbers per 10 ng DNA. (B) R. typhi^GFPuv^ bacteria were passaged weekly into fresh L929 cell cultures in 25-cm^2^ tissue culture flasks in the absence or presence of the indicated amounts of rifampin (*x* axis). DNA was prepared from the cultures after 3 and 6 passages and analyzed for the content of bacteria and the *gfp* transgene (*y* axis) by *prsA*-specific (*PrsA*) and *gfp*-specific (*GFP*) qPCR.

The growth of wild-type R. typhi was compared to that of R. typhi^GFPuv^ to assess bacterial viability. For this purpose, L929 cells were infected with equal amounts of purified wild-type or R. typhi^GFPuv^ bacteria. R. typhi^GFPuv^-infected cells were cultured in the presence of 10 ng/ml rifampin. Genomic bacterial DNA was quantified by *prsA*-specific quantitative real-time PCR (qPCR) at the indicated time points. The growth of R. typhi^GFPuv^ was comparable to that of wild-type R. typhi, with a division time of approximately 13 to 15 h (Fig. 3A). In addition, we performed *gfp*-specific qPCR to detect the transgene. As expected, the *gfp*-specific qPCR was negative for DNA derived from cultures of wild-type R. typhi, while the transgene was clearly detectable in cultures of R. typhi^GFPuv^ bacteria (Fig. 3A). An analysis of the ratio of the *gfp* transgene copy numbers to genomic copy numbers of *prsA* indicated that, on average, bacteria contained 18.5 ± 2.9 plasmid copies each. These data demonstrate that transformed R. typhi bacteria contain several copies of the plasmid, which does not affect bacterial replication and viability *in vitro*.

We further assessed retention of the plasmid in the presence or absence of rifampin. R. typhi^GFPuv^ bacteria were passaged weekly to fresh L929 cells in the presence of titrated amounts of rifampin (1, 10, or 200 ng/ml) or without antibiotics. Bacteria in the culture were monitored during this time by fluorescence microscopy and *prsA*- and *gfp*-specific qPCR. The *gfp* transgene, as well as fluorescence, was still detectable after 3 weekly passages in the absence of rifampin but was lost after 6 passages (Fig. 3B). One nanogram per milliliter rifampin in the culture medium was sufficient to maintain the plasmid, but higher copy numbers of pRAM18dRGA were present with 200 ng/ml antibiotic (Fig. 3B). These data show that maintenance of the plasmid in transformed R. typhi for more than 3 weeks depends on the presence of antibiotics.

### R. typhi^GFPuv^ bacteria are pathogenic and maintain the plasmid *in vivo*.

**FIG 4:**
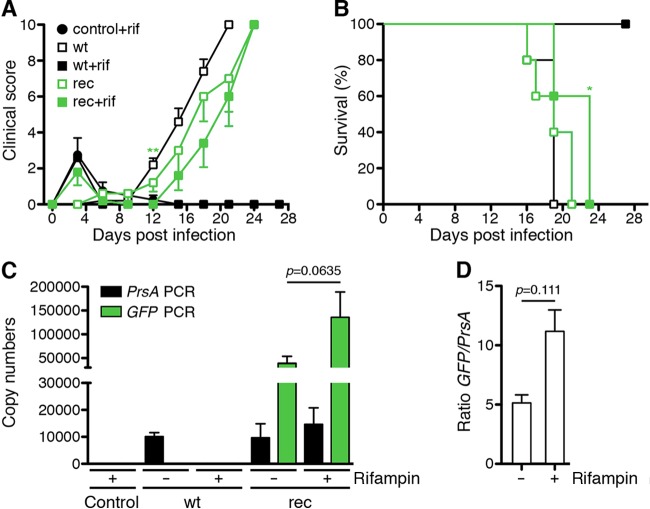
R. typhi^GFPuv^ bacteria are as pathogenic as wild-type R. typhi *in vivo*. (A) CB17 SCID mice (*n* = 5 for each group) were infected with 2 × 10^6^ SFU of wild-type R. typhi (wt) or R. typhi^GFPuv^ (rec). Control animals were not infected but received PBS. The animals were treated with rifampin (+rif) via drinking water or left untreated. The health status of the mice was monitored by a clinical score (*y* axis) at the indicated time points (*x* axis). The graph shows means ± standard errors of the mean (SEM). Statistical analysis was performed using a Mann-Whitney U test between mice infected with wild-type R. typhi without antibiotic treatment and rifampin-treated R. typhi^GFPuv^-infected animals. The asterisks indicate statistically significant differences (**, *P* < 0.01). (B) In addition, the survival rates were assessed. The survival rates of animals infected with wild-type R. typhi without antibiotic treatment and rifampin-treated R. typhi^GFPuv^-infected mice were compared using a log rank (Mantel-Cox) test. The asterisk indicates statistically significant differences (*, *P* < 0.05). (C) The bacterial loads in the spleens of the moribund animals described were determined by *prsA*-specific (black) and *gfp*-specific (green) qPCR. Organ samples from control mice and animals infected with wild-type R. typhi that were treated with rifampin and survived the infection were taken on day 30, when the experiment was terminated. The graph shows the means and SEM of the copy numbers (*y* axis) of the indicated groups of animals (*x* axis) (wt, wild-type R. typhi; rec, R. typhi^GFPuv^). (D) The ratio of *gfp* versus *prsA* copy numbers was determined for each mouse. The means and SEM of the ratio (*y* axis) in R. typhi^GFPuv^-infected CB17 SCID mice that were either untreated or received rifampin, as indicated on the *x* axis, are depicted. (C and D) Statistical analysis was performed using the Mann-Whitney U test between the indicated groups.

Having shown that R. typhi^GFPuv^ bacteria are viable and stably express the transgene *in vitro*, we further asked whether the bacteria are infectious *in vivo*. For this purpose, we employed CB17 SCID mice that succumb to infection with R. typhi within approximately 3 weeks (18). As a control, CB17 SCID mice were infected with wild-type R. typhi or received phosphate-buffered saline (PBS) instead of bacteria. The mice were treated with rifampin in drinking water as described above. Control groups of infected or PBS-treated mice did not receive antibiotics. The health status of the animals was monitored with a clinical score, and survival rates were assessed. All the animals that received antibiotics showed a weak clinical score shortly after the beginning of treatment but then recovered (Fig. 4A), most likely due to the high dose of rifampin received at the beginning of the experiment. The course of disease in R. typhi^GFPuv^-infected CB17 SCID mice treated or not with rifampin was comparable to that in animals infected with wild-type R. typhi in the absence of antibiotics, although the onset of disease was delayed by approximately 3 days. Clinical signs started to appear around day 12 in animals infected with wild-type R. typhi and around day 15 in mice infected with R. typhi^GFPuv^ (Fig. 4A, left). The mean time to death was 18 ± 1 days postinfection for animals that were infected with wild-type R. typhi and 21 ± 1 days postinfection for the group of mice that received R. typhi^GFPuv^. Rifampin-treated CB17 SCID mice that were infected with wild-type R. typhi survived the infection (Fig. 4B), demonstrating the effectiveness of the antibiotic. Thus, infection with R. typhi^GFPuv^ leads to a course and outcome of disease similar to that of infection with wild-type R. typhi, although with some delay in onset.

CB17 SCID mice develop a high bacterial load in the spleen upon R. typhi infection (18). To compare the bacterial burdens in mice infected with wild-type R. typhi and in mice infected with R. typhi^GFPuv^ and to analyze the presence of the plasmid in transgenic bacteria, we determined the bacterial content in the spleens of moribund infected CB17 SCID mice, after euthanasia, by *prsA* and *gfp* qPCR, using 20 ng DNA for each reaction. As expected, both PCRs were negative in animals of the noninfected control group (Fig. 4C). Animals that were infected with wild-type R. typhi and treated with rifampin were negative for *prsA*, except for one mouse (34.7 copies). This may indicate that in isolated cases, bacteria can persist despite antibiotic treatment. Mice infected with wild-type R. typhi that were not treated with rifampin harbored 10,085 ± 1,468 *prsA* copies in the spleen and were negative for the *gfp* transgene, as expected (Fig. 4C). CB17 SCID mice infected with R. typhi^GFPuv^ had similar *prsA* copy numbers in their spleens, whether treated with rifampin (14,662 ± 6,136 copies) or not (9,675 ± 5,206 copies) (Fig. 4C). In addition, the *gfp* transgene was detectable in both groups, with lower copy numbers in mice that were not treated with antibiotic (39,225 ± 14,365 copies) than in rifampin-treated animals (135,560 ± 53,511 copies) (Fig. 4C). On average, bacteria growing in rifampin-treated CB17 mice maintained 11.17 ± 1.81 *gfp* copies per particle, while those in nontreated animals retained only 5.12 ± 0.69 *gfp* copies each (Fig. 4D).

These data show that R. typhi^GFPuv^ bacteria are pathogenic and maintain the plasmid *in vivo* for at least 3 to 4 weeks in both the absence and presence of antibiotic selection.

### R. typhi^GFPuv^ and wild-type R. typhi bacteria induce comparable pathologies in CB17 SCID mice.

**FIG 5:**
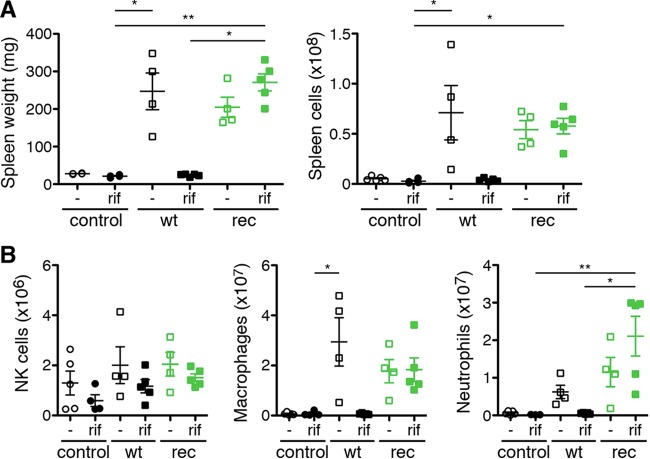
R. typhi^GFPuv^-infected CB17 SCID mice develop splenomegaly comparable to that of animals infected with wild-type R. typhi. CB17 SCID mice infected with R. typhi^GFPuv^ or wild-type R. typhi without antibiotic treatment developed splenomegaly, indicated by increased spleen weight and cell count (A). (B) NKp46^+^ NK cells were generally not significantly enhanced (left), while increased numbers of both CD11b^+^ GR1^low^ macrophages (middle) and CD11b^+^ GR1^high^ neutrophils (right) were observed. Each symbol represents a single mouse. In addition, means ± standard errors of the means (SEM) are shown. Statistical analysis was performed using one-way ANOVA (Kruskall-Wallis test followed by Dunn′s posttest). The asterisks indicate statistically significant differences (*, *P* < 0.05; **, *P* < 0.01).

Upon R. typhi infection, CB17 SCID mice develop splenomegaly due to disproportionate accumulation of macrophages and neutrophils and severe liver necrosis caused by infiltrating neutrophils (18). Therefore, we further analyzed pathology in CB17 SCID mice infected with R. typhi^GFPuv^ and wild-type R. typhi. Noninfected control mice and R. typhi-infected control mice that received rifampin and survived were euthanized at the end of the experiment (day 30). All the infected mice except those that received wild-type R. typhi and were treated with rifampin developed splenomegaly, as demonstrated by enhanced spleen weight and cell count (Fig. 5A). Thus, the treatment with rifampin generally did not affect pathology in R. typhi^GFPuv^-infected mice. Analysis of the cellular composition further revealed a comparable increase in macrophages and neutrophils in the spleens of nontreated R. typhi-infected and R. typhi^GFPuv^-infected mice, while numbers of NK cells were generally only slightly increased in infected animals (Fig. 5B).

**FIG 6:**
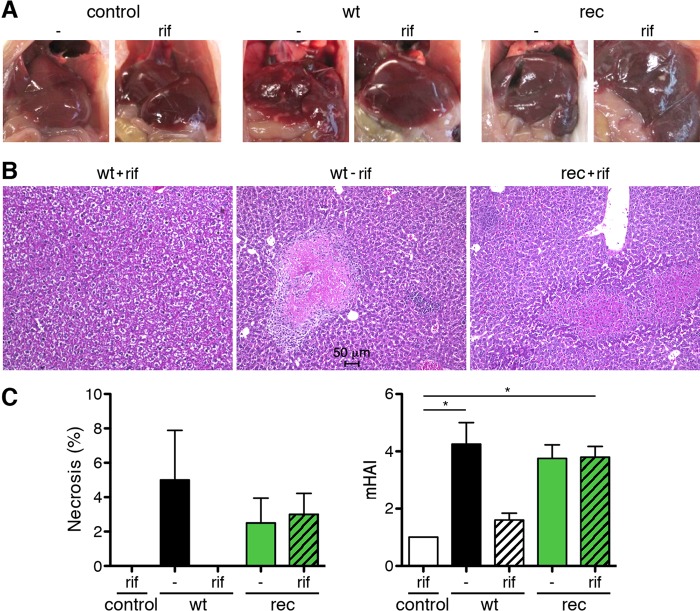
Comparable liver pathology in R. typhi^GFPuv^- and wild-type R. typhi-infected CB17 SCID mice. (A) Representative images of the livers of the animals shown in Fig. 4 and harvested from moribund mice after euthanasia demonstrate liver necrosis in mice infected with wild-type R. typhi in the absence of antibiotics and in animals that were infected with R. typhi^GFPuv^, whether treated with rifampin (rif) or not (−). Livers of control mice that received PBS or were infected with wild-type R. typhi and were treated with rifampin appeared healthy. (B) Representative H&E staining of the liver of a rifampin-treated mouse infected with wild-type R. typhi (left), a mouse infected with wild-type R. typhi without antibiotic treatment (center), and a rifampin-treated mouse infected with R. typhi^GFPuv^ (right). Livers from rifampin-treated animals infected with wild-type R. typhi appeared healthy, while necrotic lesions and cellular infiltrates were detectable in the livers from untreated mice infected with wild-type R. typhi, as well as in mice infected with R. typhi^GFPuv^. (C) The percentages of necrotic areas (left) and the mHAI were quantified. The graphs show means and SEM. Statistical analysis was performed using one-way ANOVA (Kruskall-Wallis test followed by Dunn's posttest). The asterisks indicate statistically significant differences (*, *P* < 0.05).

In addition, liver pathology was analyzed. The images in Fig. 6A and the stained histological sections in Fig. 6B show that R. typhi^GFPuv^-infected animals develop liver necrosis similar to that in CB17 SCID mice infected with wild-type R. typhi, while the livers of animals that received wild-type R. typhi but were treated with rifampin appeared healthy (Fig. 6A and B). Approximately 3 to 5% of liver tissue had undergone necrosis in wild-type R. typhi- as well as in R. typhi^GFPuv^-infected mice (Fig. 6C, left). Liver pathology was further quantified by determination of the modified hepatitis activity index (mHAI), according to the method of Ishak et al. (19). Wild-type R. typhi- and R. typhi^GFPuv^-infected mice developed comparable mHAI scores (Fig. 6C, right). These data show that R. typhi^GFPuv^ bacteria induce pathology comparable to that induced by wild-type R. typhi.

### Detection of R. typhi^GFPuv^ in organs from infected CB17 SCID mice by immunofluorescence staining.

Having shown that R. typhi^GFPuv^ bacteria are detectable by flow cytometry in L929 cells (Fig. 2), we further examined spleen cells from moribund R. typhi^GFPuv^-infected CB17 SCID mice using flow cytometry. Unfortunately, the bacteria were not detectable by this method after fixation in 2% PFA. This indicates higher sensibility of rickettsiae in spleen cells than of those in L929 cells for fixation, leading to loss of the GFP fluorescence of the bacteria. Another explanation could be a smaller number of intracellular bacteria in spleen cells than in L929 cells.

**FIG 7:**
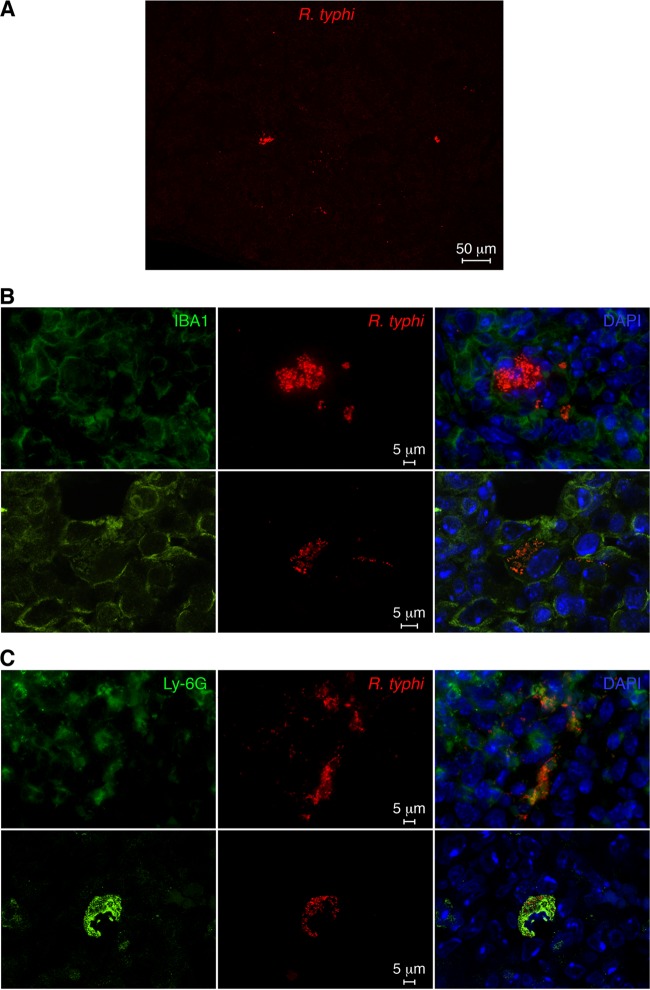
Detection of R. typhi^GFPuv^ in histological sections of the spleen from a moribund infected CB17 SCID mouse. (A) R. typhi^GFPuv^ bacteria were detected in spleen sections from a moribund infected CB17 SCID mouse by staining with anti-GFP antibody (red). An overview image is shown. (B) Sections were further stained with DAPI (nucleus; blue) and for IBA1 (green). Shown are confocal images (bottom) and overlays (right). (C) In addition, sections were stained with DAPI (blue) and for Ly-6G (green). Shown are confocal images (bottom) and overlays (right).

**FIG 8:**
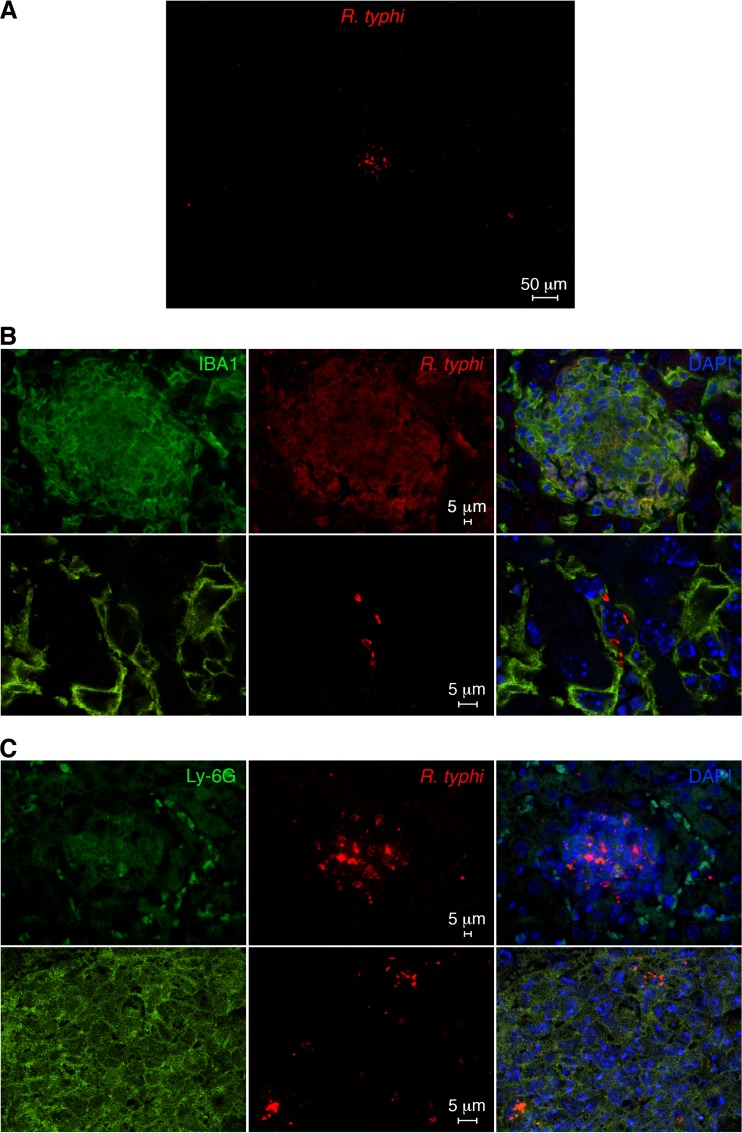
Detection of R. typhi^GFPuv^ in histological sections of liver from a moribund infected CB17 SCID mouse. (A) R. typhi^GFPuv^ bacteria were detected in liver sections from a moribund infected CB17 SCID mouse by staining with anti-GFP antibody (red). An overview image is shown. (B) Sections were further stained with DAPI (nucleus; blue) and for IBA1 (green). Shown are confocal images (bottom) and overlays (right). (C) In addition, sections were stained with DAPI (blue) and for Ly-6G (green). Shown are confocal images (bottom) and overlays (right).

We further prepared histological sections from the spleens and livers of R. typhi^GFPuv^-infected CB17 SCID mice. Because the GFP fluorescence is lost after fixation protocols, we stained the bacteria with polyclonal goat anti-GFP primary and Alexa 555-labeled secondary antibodies. In addition, sections were stained for IBA1 (macrophages) and Ly-6G (neutrophils) and Alexa 488-conjugated secondary antibodies. The bacteria were found in foci in the spleen, as well as in the liver, as shown in the overview images in Fig. 7A and 8A. Higher-resolution images and costaining for Ly-6G and IBA1 revealed that the bacteria resided in neutrophils and macrophages (Fig. 7B and C and 8B and C). Thus, the bacteria are readily detectable by immunofluorescence microscopy with the use of anti-GFP antibody staining.

### Detection of R. typhi-specific CD8^+^ T cell responses by GFP-specific restimulation.

Finally, we asked whether R. typhi^GFPuv^ bacteria can be used for the detection of specific CD8^+^ T cell responses in immunocompetent mice. Measurement of specific CD8^+^ T cell responses in rickettsial infections is difficult. MHC-I epitopes for R. typhi that could be used for CD8^+^ T cell-specific restimulation are unknown. GFP expressed by recombinant R. typhi in infected mice may have access to the MHC-I presentation pathway in infected cells to induce GFP-specific CD8^+^ T cells. If this is the case, GFP can be used as a surrogate antigen to measure R. typhi-specific CD8^+^ T cell responses.

**FIG 9:**
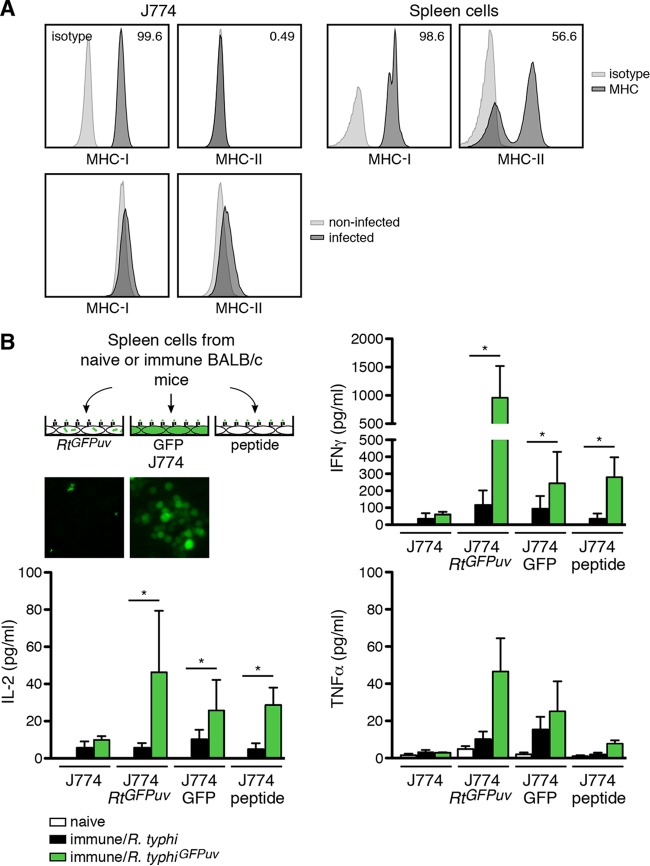
GFP-specific CD8^+^ T cells from R. typhi^GFPuv^-infected mice produce IFN-γ, IL-2, and TNF-α. (A) J774 cells were used as antigen-presenting cells in GFP-specific restimulation assays. (Top left) J774 cells were stained for extracellular MHC-I and MHC-II. (Top right) As a control for the antibody staining, spleen cells from BALB/c mice were used. (Bottom) In addition, J774 cells were infected with R. typhi^GFPuv^ and analyzed for MHC-I and MHC-II expression. J774 cells do not express MHC-II and minimally upregulate MHC-II upon R. typhi infection. (B) Different approaches were used for the restimulation of spleen cells from R. typhi-infected BALB/c mice. J774 cells were either infected with R. typhi^GFPuv^ or transduced to express cytosolic GFP 2 days prior to the assay or pulsed with GFP_200–208_ peptide 2 h before addition of spleen cells. The insets show examples of fluorescence images from R. typhi^GFPuv^-infected and GFP-expressing J774 cells, as they were used for the assay. BALB/c mice (*n* = 3) were infected s.c. with 1 × 10^6^ SFU wild-type R. typhi or R. typhi^GFPuv^. Control animals received PBS (naive). Spleen cells were isolated on day 7 postinfection; 2 × 10^5^ cells were cocultured with 4 × 10^5^ J774 cells, treated as described above or left untreated (*x* axis), in 96-well round-bottom plates in RPMI 1640 cell culture medium with 5% FCS. Cytokines were quantified in the supernatants using the Legendplex assay 48 h after the addition of spleen cells. Shown are means and SEM of the concentrations of IFN-γ, TNF-α, and IL-2 (*y* axis). Other cytokines were not detectable. Statistical analysis was performed by one-way ANOVA (Kruskall-Wallis test, followed by Dunn's posttest). The asterisks indicate statistically significant differences (*, *P* < 0.05).

We established a system for the specific restimulation of GFP-specific CD8^+^ T cells using J774 macrophage-like cells as antigen-presenting cells. Flow cytometric analysis of these cells showed that they express large amounts of MHC-I, while surface MHC-II is nearly absent (Fig. 9A, top). Furthermore, J774 cells upregulate neither MHC-II nor MHC-I upon R. typhi infection (Fig. 9A, bottom). Thus, a major contribution of CD4^+^ T cells to the observed response can be excluded. For the analysis of GFP-specific CD8^+^ T cell responses, we used different approaches, as depicted in Fig. 9B: (i) J774 cells that were infected with R. typhi^GFPuv^, (ii) J774 cells that were transduced with LeGO-G2 plasmid to express cytosolic GFP, and (iii) J774 cells that were pulsed with a GFP peptide (HYLSTQSAL; eGFP_200–208_) that had previously been identified as a H2-K^d^ CD8^+^ T cell epitope (20). Control wells contained untreated J774 cells. Spleen cells from BALB/c mice infected with either wild-type R. typhi or R. typhi^GFPuv^ were isolated on day 7 postinfection and incubated with these J774 cells. Spleen cells from naive animals were used as a control. Spleen cells from both R. typhi- and R. typhi^GFPuv^-infected mice produced small amounts of IFN-γ and IL-2 when cocultured with untreated J774 cells without further restimulation (Fig. 9B). This may be in part ascribed to CD4^+^ T cells that were also present in the culture. As expected, the response of immune spleen cells from wild-type R. typhi-infected BALB/c mice was not significantly altered in the presence of GFP-expressing or GFP peptide-pulsed J774 cells. Moreover, the presence of R. typhi^GFPuv^-infected J774 cells did not further enhance spleen cell responses (Fig. 9B), indicating that CD8^+^ T cell stimulation may be generally weak. However, immune spleen cells from R. typhi^GFPuv^-infected BALB/c mice produced significantly enhanced levels of IFN-γ in the presence of R. typhi^GFPuv^-infected J774 cells, as well as upon exposure to LeGO-G2-transduced GFP-expressing J774 cells and GFP peptide-pulsed J774 cells. The same was true for the production of IL-2, and the release of TNF-α showed the same trend (Fig. 9B). The largest amounts of these cytokines were detectable in the cultures with R. typhi^GFPuv^-infected J774 cells (Fig. 9B).

These results indicate high processing of R. typhi-derived GFP and show for the first time that transformed rickettsiae can be used for the analysis of specific CD8^+^ T cell responses.

## DISCUSSION

In the present study, we generated recombinant-GFP-expressing R. typhi (R. typhi^GFPuv^) by transformation with the plasmid pRAM18dRGA. The bacteria stably express the transgene in the presence of antibiotics and can be easily detected by flow cytometry, fluorescence microscopy, and, after anti-GFP signal enhancement, in histological sections from infected mice. R. typhi^GFPuv^ bacteria do not lose pathogenicity and induce fatal disease with kinetics and pathology similar to those of wild-type R. typhi in CB17 SCID mice. The slight delay in the onset of disease and death of the mice infected with R. typhi^GFPuv^ compared to mice infected with wild-type R. typhi was not necessarily due to the presence of the plasmid but may reflect interbatch differences. Moreover, employing R. typhi^GFPuv^ and GFP peptide-specific restimulation, we demonstrated for the first time induction of an R. typhi-specific CD8^+^ T cell response that was characterized by the release of IFN-γ, in addition to small amounts of TNF-α and IL-2.

In contrast to the genomic integration of a fusion construct of the *rpoB* promoter and *gfp_uv_* into R. typhi, which leads to reduced bacterial growth (21), the transformation of R. typhi with pRAM18dRGA, a plasmid that replicates extrasomally, did not affect the replication of the bacteria *in vitro*. The same plasmid has also been successfully used for the transformation of R. bellii, R. parkeri, R. monacensis, R. montanensis (15), R. prowazekii (17), and R. conorii (16).

R. conorii maintained the plasmid over 9 days of culture in the absence of antibiotics, although a loss of copy numbers was recognized (3.09 ± 0.52 on day 3 and 0.55 ± 0.14 on day 9) (16), while the plasmid was maintained in R. monacensis for more than 9 passages *in vitro* without antibiotic treatment (22). R. prowazekii maintained pRAM18dRGA over 10 passages with antibiotic selection (17). For R. typhi, we found that 1 ng/ml rifampin in the culture medium was sufficient to maintain the plasmid over at least 6 passages, whereas in the absence of rifampin, the plasmid was still present after 3 passages but was lost after 6 passages, demonstrating the need for antibiotic selection for culture of these bacteria, similar to R. prowazekii. Interestingly, the plasmid was maintained at a rifampin concentration much lower than the MIC for R. typhi (60 to 125 ng/ml) (23, 24), showing that the resistance gene conferred a growth advantage at rifampin concentrations lower than the MIC.

Spontaneous mutation of the *rpoB* gene, which encodes the beta subunit of bacterial DNA-dependent RNA polymerase, has been demonstrated to lead to spontaneous resistance of R. typhi to rifampin (25). Therefore, we cannot exclude the presence of rifampin-resistant bacteria lacking the plasmid in the culture. However, the copy numbers of *gfp* per bacterium did not change significantly over time in growth curve analyses in rifampin-treated *in vitro* cultures, as well as in rifampin-treated R. typhi^GFPuv^-infected mice. These observations argue against a major contribution of such a population to bacterial growth and for loss of the plasmid without antibiotic selection.

Plasmids that naturally occur in rickettsiae are in the range of 2.4 to 9.2 copies per bacterium, as demonstrated for R akari, R. bellii, and R. rhipicephali in arthropods (14). The average pRAM18dRGA plasmid copy number per R. typhi bacterium was determined to be 18.5, which is above the range of naturally occurring plasmids and in the upper range of pRAM18dRGA copy numbers that have been described in other transformed rickettsiae. The highest copy numbers were found in R. belli transformed with pRAM18dRGA (13.3 to 28.1), followed by R. parkeri (9.9), R. montanensis (7.5), and R. monacensis (5.5) (15), while plasmid copy numbers in R. conorii (1–3) (16) and R. prowazekii (0.86) were relatively low (17). These observations demonstrate that R. typhi is capable of acquisition and maintenance of much higher numbers of the plasmid than its closest relative, R. prowazekii, and SFG rickettsiae, such as R. conorii.

R. conorii maintained the plasmid during infection of susceptible C3H/HeN mice *in vivo* in the absence of antibiotic treatment (16). However, C3H/HeN mice infected with transformed R. conorii died very early after infection, between day 3 and day 5 (16). To analyze the pathogenicity of R. typhi^GFPuv^ and the maintenance of pRAM18dRGA *in vivo*, we infected CB17 SCID mice, a well-established murine model of fatal R. typhi infection. These animals succumb to infection with R. typhi within approximately 3 weeks (18). Because this is a much longer time and because we observed the loss of the plasmid in the absence of rifampin during *in vitro* culture, we decided to treat R. typhi^GFPuv^-infected animals with the antibiotic via their drinking water. In this setup, R. typhi^GFPuv^ induced a course of disease similar to and pathology comparable to those of CB17 SCID mice that had received wild-type R. typhi in the absence of antibiotic. The antibiotic dose used effectively prevented disease, including pathological changes in livers and spleens, and mortality in rifampin-treated CB17 SCID mice infected with wild-type R. typhi. Furthermore, the bacteria were detectable in spleen sections from these animals by immunofluorescence microscopy and were found in macrophages and neutrophils. These findings are in line with our previous observations employing a monoclonal R. typhi-specific antibody for flow cytometry and patient serum for the detection of the bacteria in histological sections (18).

Finally, we asked whether R. typhi^GFPuv^ can be used to investigate specific CD8^+^ T cell responses in R. typhi-infected mice. Genetically modified intracellular pathogens have been successfully employed for the detection of CD8^+^ T cell responses using the expression of a transgene as a surrogate model antigen. So far, predominantly transgenic pathogens expressing chicken ovalbumin (OVA) have been used for this purpose, as transgenic mice expressing OVA-specific T cell receptors (TCRs) are available and allow the analysis of infection-induced T cell responses. For example, the expression of exported cytosolic OVA by Plasmodium berghei ANKA has been shown to induce OVA-specific CD8^+^ T cells in infected C57BL/6 mice. These T cells act cytotoxic and may also be involved in brain pathology in cerebral malaria (26, 27). Also, OVA-transgenic intracellular bacteria, such as Listeria monocytogenes, have been used to study T cell immunology. OVA-expressing L. monocytogenes induces OVA-specific cytotoxic CD8^+^ cells in C57BL/6 mice (28, 29), and it has further been shown that cross-priming by dendritic cells (DCs) is necessary for the induction of OVA-specific CD8^+^ T cells in these animals (29). GFP expressed in the cytosol of R. typhi may similarly have access to the MHC-I presentation pathway or to cross-presentation. Indeed, we observed significant IFN-γ release by spleen cells from R. typhi^GFPuv^-infected BALB/c mice upon restimulation in the presence of R. typhi^GFPuv^-infected J774 cells, as well as lentivirus-transduced GFP-expressing J774 cells. Because J774 cells express very little MHC-II, a major contribution of CD4^+^ T cells to this response is unlikely. By using J774 cells pulsed with an MHC-I-restricted GFP peptide for restimulation of splenocytes from R. typhi^GFPuv^-infected mice, we unequivocally demonstrated that a GFP-specific CD8^+^ T cell response was induced and could be measured. To our knowledge, this is the first report employing genetically modified rickettsiae for the detection of rickettsia-specific CD8^+^ T cell responses. The detection of GFP-specific CD8^+^ T cells may be improved by rechallenge of the mice with R. typhi^GFPuv^, as has been described for OVA-expressing L. monocytogenes. In this study, rechallenge of mice with OVA-transgenic L. monocytogenes led to strongly enhanced expansion of OVA-specific CD8^+^ T cells (28). Another possibility to improve the detection of infection-induced CD8^+^ cells is the use of GFP-specific CD8^+^ T cells. A mouse strain generating GFP-specific CD8^+^ T cells has recently been described (30). CD8^+^ T cells from these animals react to GFP peptide in the context of H2-K^d^, and approximately half of the CD8^+^ T cells in these animals recognize the GFP_200–208_ epitope (30) that we also used in our study. Furthermore, GFP_200–208_/H-2K^d^ pentamers are commercially available, allowing easy detection of GFP-specific CD8^+^ T cells. Thus, BALB/c and CB17 SCID mice represent ideal models for further study of the CD8^+^ T cell response to R. typhi when using infection with R. typhi^GFPuv^, together with the methods described above. These models will also be helpful in studying trafficking of R. typhi-specific CD8^+^ T cells *in vivo* and in analyzing the interaction of specific CD8^+^ T cells with infected cells *in vitro*.

In summary, we generated transgenic R. typhi bacteria expressing GFPuv by transformation with pRAM18dRGA. These bacteria represent a tool useful not only for live-cell imaging, but also for the study of R. typhi infection *in vivo* and the analysis of R. typhi-specific CD8^+^ T cell responses.

## MATERIALS AND METHODS

### Ethics statement.

All experimentation and procedures were performed according to the German Animal Welfare Act and approved by the responsible authority (Public Health Authorities [Amt für Gesundheit und Verbraucherschutz], Hamburg, Germany; no. 33/16).

### Mice.

CB17 SCID mice were bred in the animal facilities of the Bernhard Nocht Institute for Tropical Medicine and housed in a biosafety level 3 facility for experimentation. The facilities are registered with the Amt für Gesundheit und Verbraucherschutz, Hamburg, Germany.

### Culture and purification of R. typhi.

The Wilmington strain of R. typhi was used for all experiments. γ-Irradiated (1,966 rad) L929 cells (6 × 10^6^) in 25-cm^2^ cell culture flasks were infected with the bacteria. For the preparation of bacterial stocks, cells were harvested 1 week postinfection. The cells were resuspended in 1.5 ml RPMI 1640 cell culture medium and vortexed thoroughly for 1 min with 200 μl sterile silica particles (60/90 grit silicon carbide; Lortone Inc., Mukilteo, WA, USA). The supernatant was passed through a 2-μm-pore-size filter (Puradisc 25 syringe filter; 2 μm; GE Healthcare Life Sciences, Freiburg, Germany). Bacteria were collected by centrifugation of the flowthrough at 7,826 × *g* (5 min; 4°C), washed, and resuspended in 250 or 300 mM aqueous sucrose for electroporation. For the preparation of stocks of wild-type R. typhi and transformants, the bacteria were resuspended in cell culture medium supplemented with 50% fetal calf serum (FCS) and 7.5% dimethyl sulfoxide (DMSO) and frozen in liquid nitrogen. For the infection of mice and cell cultures, bacterial stocks were thawed and centrifuged at 7,826 × *g*. The bacteria were used directly after washing in PBS. The bacterial content of the stocks was determined by qPCR. Spot-forming units (SFU) were determined by immunofocus assay, as previously described (31).

### Electroporation conditions and DNase protection assay.

Fifty microliters of bacterial suspension in 250 or 300 mM aqueous sucrose was mixed with 0.25 or 2.4 μg pRAM18dRGA plasmid DNA and placed for 15 min on ice in a 2-mm-gap electroporation cuvette (Bio-Rad, Munich, Germany). Electroporation was performed with 10 to 25 kV/cm, 10 μF, and 600 Ω, employing a Bio-Rad Micropulser apparatus (Bio-Rad, Munich, Germany). Afterward, 1 ml SPG buffer (32) was added. DNA uptake by R. typhi was determined by DNase protection assay according to the method of Binet and Maurelli (33). In brief, R. typhi bacteria were mixed with 0.25 μg of a linear double-stranded DNA (dsDNA) fragment of 2,100 bp, including the *gfp* sequence, in 250 mM aqueous sucrose in electroporation cuvettes and chilled on ice. Electroporation was performed under the indicated conditions. Electroporated suspensions were washed twice in PBS and incubated 4 times with 0.5 mg/ml DNase I (Roche, Basel, Switzerland) for 15 min each time at 37°C, with washes in between. Afterward, DNA was extracted. R. typhi copies were quantified by amplification of genomic *ompB*, as described previously (34). DNA uptake was quantified by *gfp*-specifc qPCR. To determine R. typhi survival after electroporation, L929 cell cultures in 24-well plates were infected with R. typhi suspensions that were electroporated under the indicated conditions. Samples were harvested immediately or 5 days postinfection to assess bacterial replication. These data are presented in Fig. S1 in the supplemental material. For the initial amplification of recombinant R. typhi^GFPuv^, electroporated bacteria were used to infect nonirradiated L929 cells in 25-cm^2^ cell culture flasks in RPMI 1640 medium supplemented with 5% FCS and 2 mM l-glutamine. The cells were centrifuged at 800 rpm for 30 min and then incubated at 37°C. Rifampin (10 ng/ml; Sigma-Aldrich, Munich, Germany) was added after 24 h, and subcultures were made every 3 or 4 days. Recombinant-GFPuv-expressing R. typhi (here referred to as R. typhi^GFPuv^) in infected L929 cells were detected by employing a Keyence BZ9000 microscope (Keyence, Neu-Isenburg, Germany). R. typhi^GFPuv^ bacteria were further cultured in γ-irradiated L929 cells.

### In vitro replication of R. typhi^*eGFVuv*^ versus wild-type R. typhi.

Copy numbers of bacterial stocks of purified recombinant GFPuv-expressing R. typhi and wild-type R. typhi were determined by qPCR. To compare the replication dynamics of recombinant versus wild-type R. typhi, 2 × 10^6^ L929 cells in 6-well plates were inoculated with 1 × 10^5^ copies of the respective strain in 400 μl RPMI 1640 cell culture medium supplemented with 10% FCS. The bacteria were centrifuged onto the confluent cell layer at 800 rpm for 30 min. The cells were washed with cell culture medium to eliminate nonadherent bacteria and cultivated in medium without antibiotics or containing 10 ng/ml rifampin. Cells were harvested on days 0, 1, 3, 6, and 9 postinfection. DNA was prepared, and qPCR was performed to quantify the bacterial content.

### DNA preparation from purified bacteria, cell cultures, and organs.

DNA was prepared from purified bacteria, cell cultures, and organs by employing a QIAamp DNA minikit (Qiagen, Hilden, Germany) according to the manufacturer's instructions.

### qPCR.

R. typhi genome copies were quantified either by an *ompB*-specific panrickettsia qPCR in a LighCycler 480 (Roche, Basel, Switzerland) using Hot Start *Taq* polymerase (Qiagen, Hilden, Germany) (34) or by a *prsA*-specific qPCR employing the Rotor-Gene SYBR green PCR kit (Qiagen, Hilden, Germany) in a Rotor Gene 6000 (Qiagen, Hilden, Germany), as recently described (31). Similarly, recombinant R. typhi was detected with *gfp*-specific primers (forward, 5′-CAGTGGAGAGGGTGAAGGTGATGC-3′; reverse, 5′-ACCATAAGAGAAAGTAGTGACAAGTGTTGGC-3′) under the following conditions: 15 min at 95°C, followed by 45 cycles of 10 s at 94°C, 15 s at 58°C, and 20 s at 72°C. pRAM18dRGA plasmid DNA was used as a standard.

### Flow cytometry.

R. typhi^GFPuv^-infected L929 cells were fixed in PBS-2% PFA, Cytofix/Cytoperm solution (BD Bioscience, Heidelberg, Germany), or FoxP3 staining buffer (eBioscience, Frankfurt, Germany), according to the manufacturer′s protocols, or in formaldehyde-methanol. The cells were first incubated in 1.5% formaldehyde for 10 min at room temperature, followed by incubation in ice-cold methanol for 10 min at 4°C. The cells were washed in PBS-5% FCS and analyzed with an LSRII flow cytometer using a 405-nm filter for excitation and a 530/30-nm filter for emission (BD Bioscience, Heidelberg, Germany). Noninfected L929 cells were used as a control. The expression of MHC-I and MHC-II on the surfaces of J774 cells was analyzed by staining with anti-mouse MHC-II I-A/E-phycoerythrin (PE) (1:50; clone M5/114.15.2) and anti-mouse MHC-I H2-k^d^-PE (1:50; clone SF1-1.1.1; eBioscience, Frankfurt, Germany). Rat IgG2a κ-PE (1:50; clone RTK2758; BioLegend, London, United Kingdom) was used as an isotype control. The cells were incubated with antibodies for 30 min at 4°C, washed in PBS-5% FCS, and fixed in PBS-1% PFA. Spleen cells from a BALB/c mouse were used as a staining control. The cells were analyzed with an Accuri C6 cytometer (BD Bioscience, Heidelberg, Germany). NK cells in spleen cells from infected and control mice were stained with anti-mouse NKp46-PE (clone 29A1.4; 1:200; eBioscience, Frankfurt, Germany). Macrophages and granulocytes were differentiated by staining with anti-CD11b^−^ peridinin chlorophyll protein (PerCp)-Cy5.5 (clone M1/70; 1:200; BD Bioscience, Heidelberg, Germany) and anti-GR1 (L6G/Ly6C)-allophycocyanin (APC) (clone GR-1/RB6-8C5; 1:500; Biolegend, London, United Kingdom). The cells were analyzed with an LSRII flow cytometer (BD Bioscience, Heidelberg, Germany). Macrophages were defined as CD11b^+^ GR1^low^ and neutrophils as CD11b^+^ GR1^high^.

### Infection of CB17 SCID and BALB/c wild-type mice.

Purified R. typhi^GFPuv^ or wild-type R. typhi bacteria (2 × 10^6^ SFU) were injected into BALB/c or CB17 SCID mice subcutaneously (s.c.) at the base of the tail. Infected animals received drinking water without antibiotics or supplemented with rifampin. The average weight of the mice was 20 g at the beginning of the experiment, and the average drinking water consumption per animal was approximately 3 ml per day. Rifampin was added as follows: infected CB17 SCID mice initially received 100 mg/kg of body weight/day for the first 3 days and then 10 mg rifampin/kg/day. Drinking water was changed every 2 or 3 days. The state of health of susceptible CB17 SCID mice was evaluated with clinical scores as follows: posture (0, normal; 1, temporarily hunched; 2, hunched), fur condition (0, normal; 1, staring in the neck; 2, overall ruffled), activity (0, normal; 1, reduced; 2, strongly reduced), weight loss (0, <10%; 1, 10 to 14%; 2, >20%), and food and water uptake (0, normal; 1, reduced; 2, none) (18). Mice were considered healthy to moderately ill with a score of <5, ill with a score of 5 to 7, and severely ill with a score of 8 to 10. Mice were euthanized when they reached a total score of >8 or showed weight loss of >20%. All the CB17 SCID mice were euthanized when they showed severe signs of disease. Organs were prepared for the assessment of bacterial loads and the presence of the transgene by qPCR and histology. For flow cytometric analysis of spleen cells, R. typhi^GFPuv^-infected CB17 SCID mice were euthanized on day 14 postinfection. Spleen cells from resistant R. typhi- or R. typhi^GFPuv^-infected BALB/c mice and noninfected control animals were prepared on day 7 postinfection for *in vitro* restimulation.

### Transduction and infection of J774 macrophages.

Lentiviral particles that carried the LeGO-G2 vector for the expression of enhanced GFP (eGFP) controlled by a spleen focus-forming virus (SFFV) promoter were produced in HEK293T cells according to the recommended protocol (http://www.lentigo-vectors.de). J774 macrophages were transduced in 24-well plates with 50 μl LeGO-G2 particles in the presence of 8 μg/ml Polybrene (Sigma-Aldrich, Munich, Germany). For infection with R. typhi^GFPuv^, J774 cells were seeded into 24-well plates and exposed to 10 bacteria per cell. Transduction, as well as infection, of J774 cells was performed 48 h prior to the addition of T cells.

### GFP-specific *in vitro* restimulation of spleen cells from infected BALB/c mice.

BALB/c mice were infected s.c. with 2 × 10^6^ SFU of purified R. typhi^GFPuv^ or wild-type R. typhi or treated with PBS as a control. Spleen cells were prepared on day 7 postinfection; 2 × 10^5^ spleen cells were incubated with 5 × 10^4^ J774 macrophages that were infected with R. typhi^GFPuv^, that expressed cytosolic GFP after transduction with LeGO-G2 particles, or that were untreated. In addition, cocultures of J774 macrophages and spleen cells were treated with the eGFP peptide HYLSTQSAL (0.2 μg/ml; eGFP_200–208_) that is also present in the GFPuv sequence and has been identified as an H2-K^d^ CD8^+^ T cell epitope (20). Cytokine production was analyzed 48 h after the addition of T cells by Legendplex assay (Biolegend, London, United Kingdom).

### Histology.

Spleens and livers from infected mice were fixed in PBS with 4% formalin and embedded in paraffin. Sections deposited onto slides were deparaffinized by heating at 63°C for 30 min, followed by a 30-min incubation in dimethylbenzol (Xylol). The sections were then soaked for 3 to 5 min each time in a descending series of ethanol (EtOH) concentrations (3× 100% EtOH; 3× 96% EtOH, 80% EtOH, 70% EtOH). Finally, the slides were washed in H_2_O and boiled for 30 min in 10 mM sodium citrate-0.05% Tween20 (pH 6.0) for antigen retrieval. A Ventana Benchmark XT apparatus (Ventana, Tuscon, AZ, USA) was used for staining. Antibodies were diluted in 5% goat serum (Dianova, Hamburg, Germany) in Tris-buffered saline (pH 7.6) (TBS) and 0.1% Triton X-100 in antibody diluent solution (Zytomed, Berlin, Germany). R. typhi^GFPuv^ bacteria were detected with polyclonal goat anti-GFP (1:100; Novus Biologicals, Wiesbaden, Germany) and rabbit anti-goat superclonal-Alexa 555 antibody (1:500; ThermoFisher/Life Technologies, Darmstadt, Germany). Rabbit anti-mouse IBA1 (1:300; 019-19741; Wako, Neuss, Germany) and rat anti-mouse Ly-6G (1:1,000; clone 1A8; BD Biosciences, Heidelberg, Germany) were used for the detection of macrophages and neutrophils and counterstained with Alexa 488-labeled chicken anti-rat antibody (1:200; ThermoFisher/Life Technologies, Darmstadt, Germany) or Alexa 488-labeled chicken anti-rabbit antibody (1:300; ThermoFisher/Life Technologies, Darmstadt, Germany), respectively. Slides were incubated with primary and secondary antibodies for 1 h. Nuclei were stained with DAPI (4′,6-diamidino-2-phenylindole) (1:1,000; Sigma-Aldrich, Munich, Germany). Sections were covered with Tissue-Tek embedding medium (Sakura Finetek, Staufen, Germany). Liver damage was evaluated in hematoxylin and eosin (H&E)-stained sections by the mHAI, according to the method of Ishak et al. (19).

### Fluorescence microscopy.

Cell cultures and histological sections were analyzed by fluorescence microscopy. For high-resolution images, cells were plated on 35-mm μ-dishes or 24-well μ-plates (Ibidi; Martinsried, Germany) and infected with R. typhi^GFPuv^. Images were taken with a Keyence BZ9000 microscope (Keyence, Neu-Isenburg, Germany). Confocal images were taken with a Leica SP5 confocal microscope (Leica Microsystems, Wetzlar, Germany) in the Flow Cytometry Core Facility at the Research Center Borstel, Borstel, Germany.

### Statistical analyses.

Statistical analyses were performed with Prism 5 software. Groups were compared using one-way analysis of variance (ANOVA) (Kruskal-Wallis test followed by Dunn's posttest), Mann-Whitney U test, or Mantel-Cox test as stated in the figure legends.

## Supplementary Material

Supplemental material
