# Supplementary material for: GFPuv-Expressing Recombinant Rickettsia typhi: a Useful Tool for the Study of Pathogenesis and CD8+ T Cell Immunology in R. typhi Infection
Source: Infect Immun. 2017 May 23;85(6):e00156-17. doi: 10.1128/IAI.00156-17 (PMC5442613; doi:10.1128/IAI.00156-17)
Supplement: Supplemental material [file supp_85_6_e00156-17__index.html]

GFPuv-Expressing Recombinant Rickettsia typhi: a Useful Tool for the Study of Pathogenesis and CD8+ T Cell Immunology in R. typhi Infection — Supplemental material 

# GFPuv-Expressing Recombinant Rickettsia typhi: a Useful Tool for the Study of Pathogenesis and CD8+ T Cell Immunology in R. typhi Infection

## Supplemental material

- Supplemental file 1 -

  Fig. S1. Determination of electroporation conditions: bacterial growth after electroporation and DNAse protection assay.

  PDF, 189K
