# Supplementary material for: GFPuv-Expressing Recombinant Rickettsia typhi: a Useful Tool for the Study of Pathogenesis and CD8+ T Cell Immunology in R. typhi Infection
Source: Infect Immun. 2017 May 23;85(6):e00156-17. doi: 10.1128/IAI.00156-17 (PMC5442613; doi:10.1128/IAI.00156-17)
Supplement: Supplemental material [file IAI.00156-17_zii999092035s1.pdf]

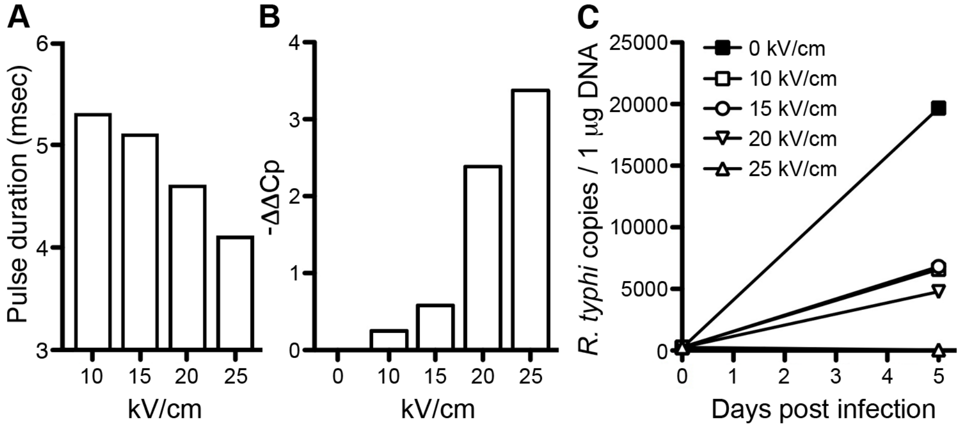

Fig. S1. Determination of electroporation conditions: Bacterial growth after electroporation and DNAse protection assay. DNA-containing *R. typhi* suspensions were electorporated with indicated field strengths (x-axis). The pulse durations for the different electroporation conditions are depicted (y-axis) (**A**). To quantify the DNA uptake by the bacteria, extracellular DNA in electroporated samples was digested by DNAse I. Copy numbers (Cp) of *R. typhi* and of *GFP* were quantified by qPCR. The ΔΔCp values (y-axis) describe the differences between the Cp values of the *R. typhi*- and *GFP*-specific qPCRs that were normalized to the 0 kV/cm values ($\Delta\Delta Cp = \Delta Cp_{GFP} - \Delta Cp_{R.\ typhi}$) (**B**). To quantify *R. typhi* survival, L929 cells were infected with the electroporated bacteria. *R. typhi* copy numbers in the cell culture (y-axis) were quantified by *OmpB*-specific qPCR at indicated points in time (x-axis) (**C**).
